# Modeling, Optimization and Performance Evaluation of TiC/Graphite Reinforced Al 7075 Hybrid Composites Using Response Surface Methodology

**DOI:** 10.3390/ma14164703

**Published:** 2021-08-20

**Authors:** Mohammad Azad Alam, Hamdan H. Ya, Mohammad Yusuf, Ramaneish Sivraj, Othman B. Mamat, Salit M. Sapuan, Faisal Masood, Bisma Parveez, Mohsin Sattar

**Affiliations:** 1 Mechanical Engineering Department, Universiti Teknologi PETRONAS, Seri Iskandar 32610, Malaysia; neishrama@gmail.com (R.S.); drothman_mamat@utp.edu.my (O.B.M.); mohsinsheikh00a@gmail.com (M.S.); 2 Chemical Engineering Department, Universiti Teknologi PETRONAS, Seri Iskandar 32610, Malaysia; yusufshaikh.amu@gmail.com; 3 Laboratory of Biocomposite Technology, Institute of Tropical Forest, and Forest Products (INTROP), Universiti Putra Malaysia, Serdang 43400, Malaysia; sapuan@upm.edu.my; 4 Advanced Engineering Materials and Composites Research Centre (AEMC), Department of Mechanical and Manufacturing Engineering, Faculty of Engineering, Universiti Putra Malaysia, Serdang 43400, Malaysia; 5 Electrical and Electronics Engineering Department, Universiti Teknologi PETRONAS, Seri Iskandar 32610, Malaysia; fslmsd@gmail.com; 6 Department of Manufacturing and Materials Engineering, International Islamic University Malaysia, Kuala Lumpur 53100, Malaysia; mirbisma5555@gmail.com

**Keywords:** Al 7075 hybrid composites, modeling and optimization, powder metallurgy, response surface methodology, microhardness, ANOVA

## Abstract

The tenacious thirst for fuel-saving and desirable physical and mechanical properties of the materials have compelled researchers to focus on a new generation of aluminum hybrid composites for automotive and aircraft applications. This work investigates the microhardness behavior and microstructural characterization of aluminum alloy (Al 7075)-titanium carbide (TiC)-graphite (Gr) hybrid composites. The hybrid composites were prepared via the powder metallurgy technique with the amounts of TiC (0, 3, 5, and 7 wt.%), reinforced to Al 7075 + 1 wt.% Gr. The microstructural characteristics were investigated by optical microscopy, scanning electron microscopy (SEM), X-ray diffraction (XRD) and energy dispersive X-ray spectroscopy (EDS) elemental mapping. A Box Behnken design (BBD) response surface methodology (RSM) approach was utilized for modeling and optimization of density and microhardness independent parameters and to develop an empirical model of density and microhardness in terms of process variables. Effects of independent parameters on the responses have been evaluated by analysis of variance (ANOVA). The density and microhardness of the Al 7075-TiC-Gr hybrid composites are found to be increased by increasing the weight percentage of TiC particles. The optimal conditions for obtaining the highest density and microhardness are estimated to be 6.79 wt.% TiC at temperature 626.13 °C and compaction pressure of 300 Mpa.

## 1. Introduction

Today’s globe is tormented by environmental pollution and fossil fuel scarcity. The automotive industry is one of the most significant contributors to these issues. Around 1.2 billion motor vehicles are anticipated to be on the road, accounting for 75% of pollution, 27% of greenhouse gas emissions, and 756 L/year of gasoline consumption [1,2,3,4]. The level of pollution and fuel consumption can be lowered considerably by increasing the vehicle’s fuel economy. One approach to enhance fuel efficiency is to make the vehicle lighter by utilizing lightweight materials like aluminum and aluminum-based hybrid composites [4]. Hence, the demand for performance aluminum composites capable of withstanding harsh engineering conditions is already rising exponentially. Vital automobile components such as drive shafts, brake discs, pistons and cylinder heads, cylinder liners experience massive structural loads and harsh conditions throughout their service operation [5]. Thus, the various interior and exterior components of automotive are susceptible to scratches or indentations and wear loss while in operation. Hence, an adequate analysis of the surface hardness behavior of aluminum matrix composites (AMCs) is therefore of pivotal importance. Significant investigations have therefore been focused on the production of AMCs due to their low density, improved strength to weight ratio, and satisfactory hardness and wear resistance [6,7,8,9,10,11,12]. These key features of Al/Al alloy composites have led to a great acceptance of AMCs in automobiles, aircraft, offshore structures, and many other applications. The governing factors for the enhanced properties of AMCs are their processing techniques, particle distribution, the alignment nature of reinforcements within the matrix, and the interaction of filler particles at interfaces [11]. In addition to these unique properties, the hardness behavior improvement of AMCs has drawn considerable interest in the automotive and aircraft industries. Recent studies have adopted various existing manufacturing techniques, such as friction stir processing [13,14,15,16], mechanical alloying [17,18,19], friction stir processing [4], stir casting [20,21,22], and powder metallurgy [23,24,25,26] to fabricate improved hardness and scratch-resistant AMCs. Interestingly, powder metallurgy (PM) has attracted increasing interest in producing AMCs due to its significant benefits, such as the uniform dispersion of reinforcement particles in the matrix and limited likelihood of forming interfacial phases owing to reduced processing temperature [27]. Furthermore, the PM method is economical in large-scale manufacturing because it minimizes expensive machining processes owing to the development of near-net-shaped components.

Optimization of the process variables is the major factor in the experimental work to save labor, materials, and money as well as to get an improved response [28]. In recent years numerous modeling and optimization tools like artificial neural networks and response surface methodology has been used by various researchers in a vast variety of experimental and simulation-based works [29]. Therefore, the latest and advanced statistical tools must be implemented within the investigation boundary conditions. Response surface method (RSM) is a highly advanced DOE technique, which uses a statistical formulation for developing a model and analyzing a process that aims to optimize the desired response controlled by multiple input factors [30,31,32,33] Using a relatively small number of experiments, a response surface model can be used to map a design space [34]. Surface and contour graphs are frequently used to explain both linear and nonlinear mixing complications of mixed components.

There are numerous studies on the effective manufacture of AMCs strengthened with a few common reinforcements such as SiC [27,34,35,36], TiC [6,18,20], B_4_C [37], TiO_2_ [38], etc. Among these, TiC is the most frequently utilized ceramic particle reinforcement to produce AMCS. TiC has a high melting point as well as increased tribological characteristics. Hence, TiC provides many exceptional features, namely high hardness, higher resistance against wear, and reasonable thermal stability. Due to these unique features, TiC is chosen as one of the fillers in the present study.

Integrating soft carbon-based particulates like graphite [39,40], graphene [41,42], and carbon nanotubes (CNTs) into hard materials produces decent self-lubricating hybrid composites that afterward can provide improved hardness and wear resistance by lowering the moving surface temperature. Recent research of this creative method has shown that the development of a consistent layer of lubricant upon this tribo-surface will minimize shear forces and permanent deformation within the subsurface area [19,43]. Therefore, it would be advantageous to use TiC as a reinforcement in the aluminum alloy matrix together with microparticles of graphite to improve the hardness behavior. In today’s of Industry 4.0 revolution era hybrid composites are widely utilized in the automotive sector. The hybrid metal matrix composite is a type of material displaying the advantages with two or more fillers inside the metal/alloy.

Various investigations were reported for the enhancement of the mechanical and tribological properties of Al-TiC and Al-graphite composites. The inclusion of hard reinforcement particles into the Al matrix through an ex-situ or an situ method can improve the hardness and strength, while strongly influencing the wear characteristics of AMCs. As indicated by previous research [28], the main downside of the incorporation of an elevated amount of reinforcements in a matrix is acute wear in counter-face elements due to the elevated hardness. As a result, the benefits of reinforcement addition must be preserved so that the strength and wear resistance of AMCs are never compromised. Thus, in present investigation 1 wt.% graphite was reinforced with Al 7075/TiC composites to improve the hardness by maintaining good wear characteristics. Also, the research related to synthesis, characterization, and in-depth study on the microhardness behavior of Al 7075-TiC-graphite hybrid composites produced by Turbula mixing followed by the powder metallurgy steps are seldom found in the literature.

In the present study, Al 7075-Gr 1 wt.%-TiC x wt.%, TiC (x = 3, 5, and 7%) sintered hybrid composites were produced via a powder metallurgy technique. A Box Behnken design (BBD) response surface methodology (RSM) approach was utilized for modeling and optimization of density and microhardness independent parameters. The developed quadratic model correlates the density and microhardness with specific process variables. Furthermore, the effect of process variables (i.e., TiC concentration, sintering temperature, compaction pressure) on the sintered density and microhardness characteristics of the produced hybrid composite has been investigated. Finally, the developed model was experimentally corroborated to confirm its accuracy for the prediction of the responses. The microstructural characteristics were investigated by Optical microscopy, scanning electron microscopy (SEM), X-ray diffraction (XRD) and energy dispersive X-ray spectroscopy (EDS) elemental mapping. This study presents considerable insights into the density and microhardness analyses of aluminum alloy matrix hybrid composites.

## 2. Materials and Methods

### 2.1. Starting Materials and Characterizations

The matrix material Al 7075 was used for the fabrication of hybrid composites. The elemental chemical composition of the Al 7075 matrix is expressed in Table 1. Spherical-shaped Al 7075 powder with an average particle size of 8–15 µm was purchased from the CNPC Powder Co. Ltd., (Shanghai, China). The reinforcements chosen for hybrid composite fabrication were titanium carbide (99.9% purity, 200–800 nm, supplied by Nova Scientific Selangor, Malaysia) and graphite (average particle size approximately 38µm, supplied by Ugent Ltd., Ipoh, Malaysia) respectively.

### 2.2. Composites Fabrication Processing

**Figure 1:**
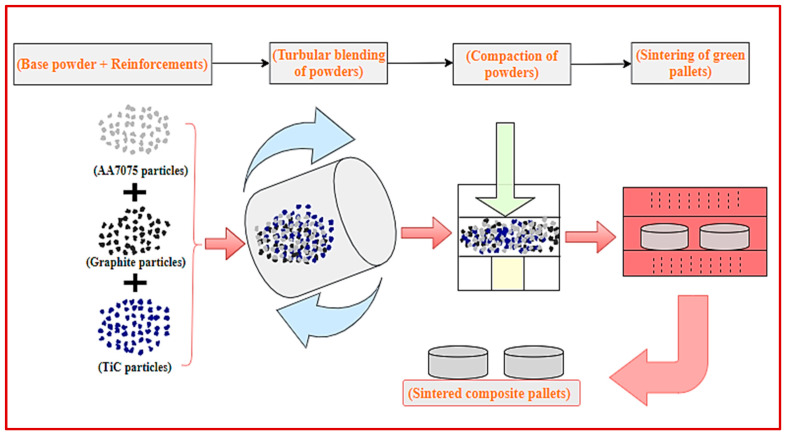
Schematic illustration of the fabrication of sintered composites through powder metallurgy.

In the present study, a green fabrication process, i.e., the powder metallurgy (PM) technique, was adopted for the synthesis of composites. Five different combinations of composites were synthesized by the PM process, as depicted in Table 2. The matrix and reinforcement powders were mixed using Turbula mixing. The schematic diagram for the synthesis of composites in various stages is illustrated in Figure 1. The main steps of producing composites were mixing elemental powders in a Turbula mixer (Shanghai, China), drying of the mixed powder in vacuum oven, compaction of powder, and sintering of compact pallets.

#### 2.2.1. Mixing of Powders and Compaction

**Figure 2:**
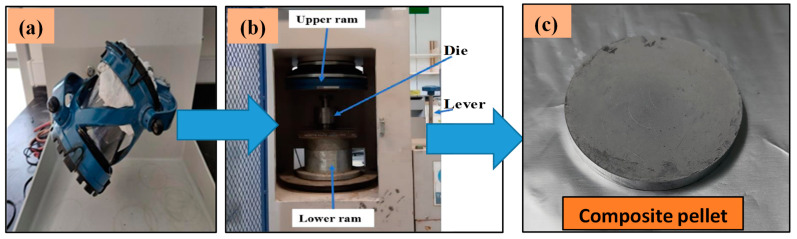
(**a**) Turbula mixer used for blending of powders; (**b**) Hydraulic press used for the uniaxial cold compaction process; (**c**) Composite pellet after compaction.

The various compositions of composite powders were blended in Turbula mixer as shown in Figure 2a for 4 h to get well homogeneous powder combinations. To formulate the hybrid composites, different wt.% (3, 5, and 7%) of TiC were added to premixed Al 7075/1 wt.% graphite composites powder. The synthesized composite powders were put in a controlled drying oven to remove moisture/impurities from the mixed composite powders.

Cold compaction (uniaxial) was carried out on all the formulations of composite powders at a compaction pressure of 250–350 MPa in a uniaxial hydraulic pa let press (ELE International, Bedfordshire, UK) as shown in Figure 2b. A mixed composite powder sample of 15 g weight was put in a die steel mold. Pressure is applied gradually to compact the powder, and the size of the pellets produced was 30 mm diameter and 8 mm thickness. Paraffin wax was applied around the die walls to avoid die friction. The green pallet after compaction is depicted in Figure 2c. The green density of all the pallets was measured before the sintering process.

#### 2.2.2. Controlled Environment Sintering

**Figure 3:**
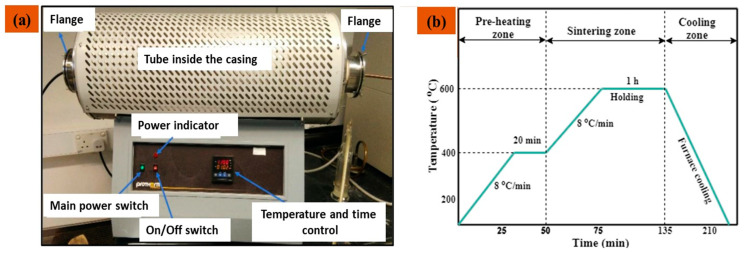
(**a**) Tube furnace utilized for sintering of the green compacts; (**b**) Schematic illustrations of Sintering cycle in controlled nitrogen atmosphere of the tube furnace.

The fabricated green pellets of Al 7075 and Al 7075/Gr/TiC hybrid composites were pressurelessly sintered in a nitrogen environment for 2.25 h in the tube furnace (Protherm, PTF12/75/800, Ankara, Turkey) as shown in Figure 3a. The heating rate was maintained a 8 °C/min, and the sintering temperature and dwell time were kept 500–600 °C and 1 h, respectively, for all samples. The cooling of samples to ambient temperature was done in the furnace as depicted in the sintering cycle illustration (Figure 3b).

### 2.3. Microstructural Characterizations (OM, XRD, EDS, and Elemental Maps)

The microstructural observations of sintered composites were examined by optical microscopy (Leica DM LM, Wetzlar, Germany), and field emission scanning electron microscopy (FESEM, Phenom-Pro X, Waltham, MA, USA and Zeiss Supra 55VP, Jena, Germany), equipped with an energy dispersive spectroscopy (EDS) attachment. Optimal microscopy observations were performed on the surface of the polished and etched (using Keller’s reagent) composite.

The X-ray diffraction (XRD) analyses of synthesized sintered composites were conducted by using an X-ray diffractometer (PANalytical X’pert, Almelo, Netherlands). The XRD observations were performed by applying Cu-Ka radiation (wavelength, λ = 0.154 nm), with operating parameters 40 kV and 40 mA. The XRD scanning speed was maintained as 1°/min with a scanning range (2θ) of 20–80°. The Highscore Plus software was used to assess the samples’ XRD patterns.

### 2.4. Density and Porosity Measurement of Composites

The green density of each produced sample was measured before the sintering process. The diameter, thickness, and mass measurements of the green compact samples were used to measure green densities for all the samples. The sintered densities of Al and composite samples were measured by utilizing an electronic densitometer (Mettler Toledo) according to Archimedes’ principle by a standard test method (ASTM B962-17). The effectiveness of the sintering process is normally represented by the sintered density. For each formulation, the average of five sample’s sintered density was reported. The sintering operation was assessed using sintered porosity and densification factor of synthesized composites as mentioned in Equations (1) and (2):

(1) $$\mathrm{Porosity}\;\left(\%\right)\;=\frac{\mathrm{Theoretical}\;\mathrm{density}-\mathrm{Sintered}\;\mathrm{density}}{\mathrm{Theoretical}\;\mathrm{density}}\times 100$$

(2) $$\;\mathrm{Densification}\;\mathrm{factor}=\frac{\mathrm{Sintered}\;\mathrm{density}-\mathrm{Green}\;\mathrm{density}}{\mathrm{Theoretical}\;\mathrm{density}-\mathrm{Green}\;\mathrm{density}\;}$$

The theoretical density of the composites is the highest density that can be achieved without void spaces as determined by the rule of mixture, based on sample formulations and pure component densities. Green density refers to the density of the green compact, which reflects the powder-to-powder contact area and aids the bonding process during sintering. It was calculated by the density formula by measuring the mass and volume of the green compacts.

### 2.5. Microhardness Measurement

The microhardness measurement for all the polished sintered composites was accomplished by utilizing a Vickers hardness tester (Leco LM 247 AT, Saint Joseph, MO, USA). The standard test method (ASTM E92-82) was followed for the measurement of microhardness. The test was conducted at ambient temperature, the indentation load was kept at 300 gf with a dwell time of 15 s. At least five microhardness values were recorded at different locations of each test sample and average values were taken into consideration.

### 2.6. Box-Behnken Experimental Design

Design of experiment (DOE) is a systematic approach for determining the worth of variables, their interactions, and controlling them to achieve the best possible outcome. The Box-Behnken design (BBD) approach was utilized under response surface methodology (RSM) to develop the mathematical models using the Design-Expert version 12 software (Stat-Ease. Inc., Minneapolis, MN, USA).

The Box-Behnken design is a factorial arrangement of at least three variables with incomplete block designs. In each block, one factor is kept constant at the center point, while the others vary based on four distinct combination values with upper and lower limits [44]. The three input parameters (sintering temperature, compaction pressure, and the content of TiC wt.%) were analyzed using 17 runs of experiments with their low and high values. The experiments were performed corresponding to a three-level scale, i.e., the lower value (−1), central value (0), and higher value (+1). The ranges and levels of independent variables are listed in Table 3. The designed experiments were carried out, and the response surface values were fed into the software. The significance of input parameters and their interaction effects on the output response variables was established using analysis of variance (ANOVA). Table 4 depicts the actual experimental design matrix as per the BBD interface along with their response.

## 3. Results and Discussion

### 3.1. Characterization of Starting Powders

**Figure 4:**
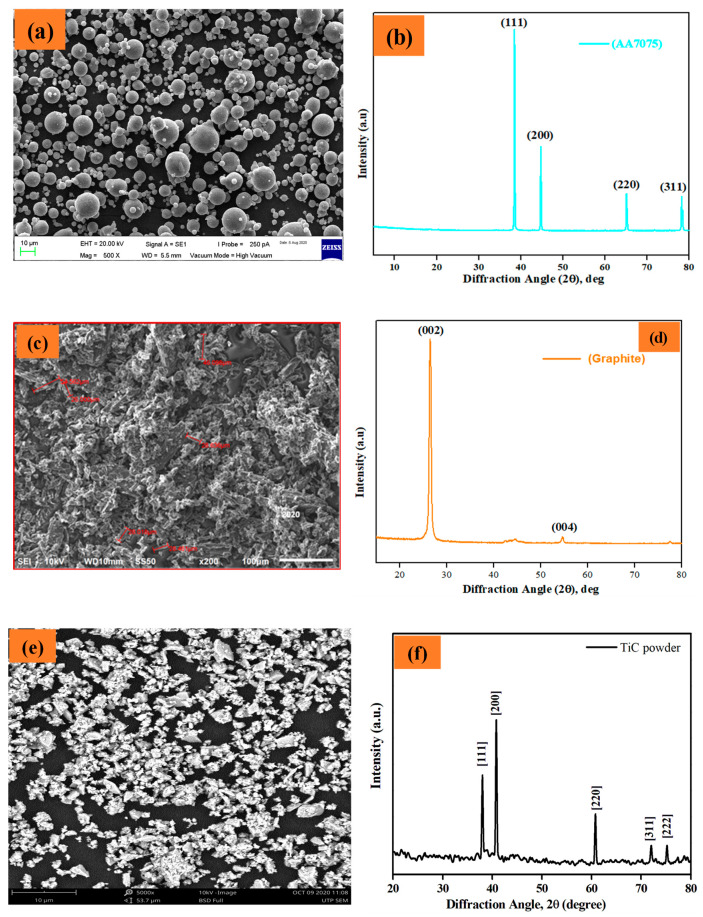
SEM micrographs and XRD peaks of as-received powder particles (**a**,**b**) Al 7075; (**c**,**d**) Graphite; (**e**,**f**) titanium carbide.

The powder morphology and X-ray diffraction peaks of the as-received pure Al 7075, particles of graphite and TiC powders are illustrated in Figure 4a–e. The Al 7075 powder particles are spherical, with few particles of irregular shape (Figure 4a). The graphite particles morphology is illustrated in Figure 4c, the particles are irregular in shape with an average particle size of approximately 38 µm. The TiC powders (<800 nm) have an irregular shape and were agglomerated (Figure 4e).

The X-ray diffraction patterns of the starting powders (Al 7075, graphite, and TiC) are illustrated in Figure 4b,d,f respectively. As observed from Figure 4b, all the major peaks of Al 7075 belonged to Al with an FCC crystal structure and identified peaks include (111), (200), (220), and (311) at a diffraction angle (2θ) = ~38°, 45°, 65° and 78°, respectively.

The phase analysis is verified by comparison with existing literature [45]. The peaks of graphite powder were identified as (002) and (004) at (2θ) = ~26° and 54°, respectively (Figure 4d). The phase analysis of graphite powder is in agreement with existing literature [46]. All five diffraction peaks of TiC (111), (200), (220), (311), and (222) were detected as belonging to TiC with cubic structure and having lattice parameter a = 4.3254 Å, also in agreement with the literature [17,47]. These phase analyses can be utilized in the phase validation with the produced composite samples.

### 3.2. Characterization of Al 7075-Graphite-TiC Hybrid Composites

The mechanical characteristics of the Al alloy-based composites are influenced not only by the weight fraction and the nature of reinforcements but also by the distribution of the reinforcement and the matrix morphology. Hence, the following characterizations are done to minutely observe the morphology, phase identification, and distribution of the reinforcements in the matrix.

#### 3.2.1. Optical Microscopy Observations

**Figure 5:**
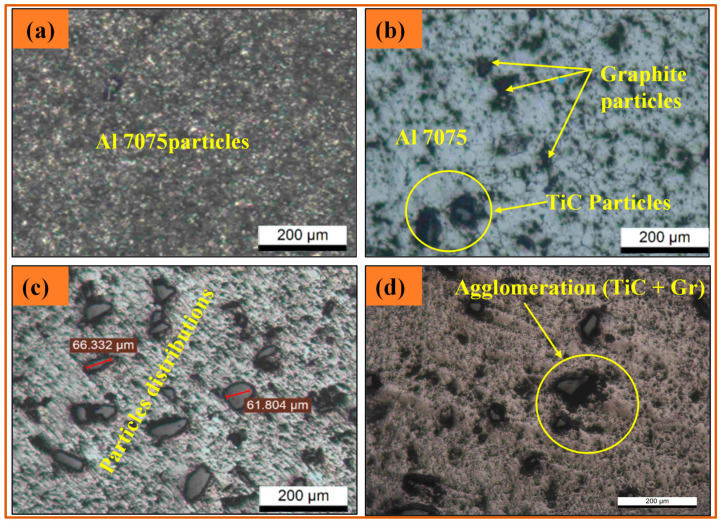
Optical microscopy observations of (**a**) Al 7075; (**b**) Al 7075 + 1 wt.% Gr + 3 wt.% TiC; (**c**) Al 7075 + 1 wt.% Gr + 5 wt.% TiC; (**d**) Al 7075 + 1 wt.% Gr + 7 wt.% TiC.

**Figure 6:**
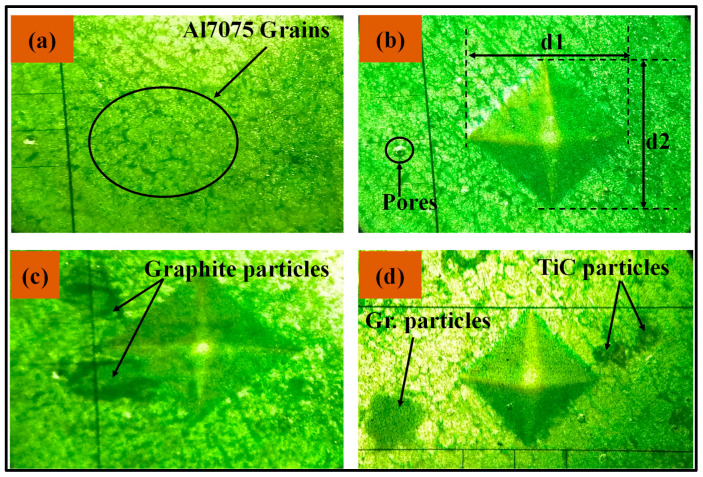
Optical micrograph of Al 7075 (**a**) before hardness test; (**b**) Indentation image after hardness test; (**c**,**d**) Indenter impact on Al 7075/Gr and Al 7075/Gr/TiC composites surface, respectively.

Figure 5 shows optical microscopy images of the matrix Al 7075 and all the synthesized composite samples. As observed from the image of Al 7075, no pores are present in the microstructure (Figure 5a). The grain boundaries are visible along with the particle distributions of the reinforcements within the matrix Al 7075 (Figure 5b). An optical micrograph of a hybrid composite with 3 wt.% TiC is shown in Figure 6c, where a slight agglomeration is observed for the graphite particles. Refinement of grains is also observed. A higher level of agglomeration is exhibited in the 7 wt.% TiC hybrid composite sample. The distributed nature of the reinforcement plays a decisive role in the enhancement of the physical and mechanical behavior of the produced composites [34].

Figure 6 illustrates the optical microscopy results of the sintered samples before and after the microhardness test. The grains and grain boundary of Al 7075 before the microhardness test are seen in Figure 6a, while Figure 6b depicts the indentation impact on the surface of the Al 7075. In Figure 6b, the d_1_ and d_2_ indicate the length of the diagonal of the indentation of the indenter. The indentation image for Al/graphite is observed in Figure 6c, the graphite particles are highlighted in the image. For the hybrid composites, Al/Gr/TiC is shown in Figure 6d, where the agglomeration of TiC particles is observed.

#### 3.2.2. XRD of Al/Gr/TiC Hybrid Composites

**Figure 7:**
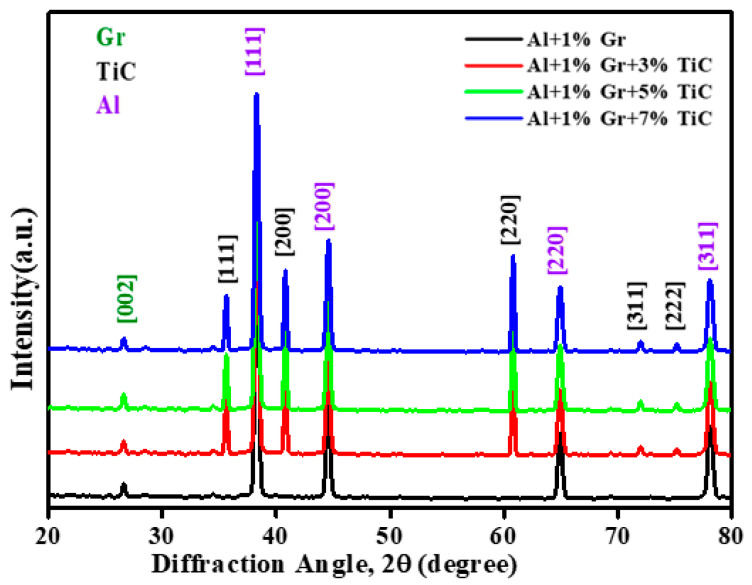
XRD peak patterns for Al/graphite composites and Al/Gr/TiC hybrid composites with 0, 3, 5 and 7 wt.% of titanium carbide and 1% graphite.

The X-ray diffractometry technique was used to determine the phase identification and confirm of the existence of reinforcing particles (Gr and TiC) in the sintered composites. Figure 7 shows the X-ray diffraction (XRD) patterns of produced Al 7075/Gr composites and Al 7075/Gr/TiC hybrid composites with varying percentages of graphite.

The presence of Al 7075, Gr, and TiC in the produced hybrid composite samples is confirmed by their respective peaks in the XRD plot indicated by blue, green, and red colored lines. The highest peak is observed in Al 7075, accompanied by TiC and graphite. The findings are consistent with previously published research [48,49,50].

#### 3.2.3. EDS Analysis of Matrix Al 7075 and Hybrid Composites

**Figure 8:**
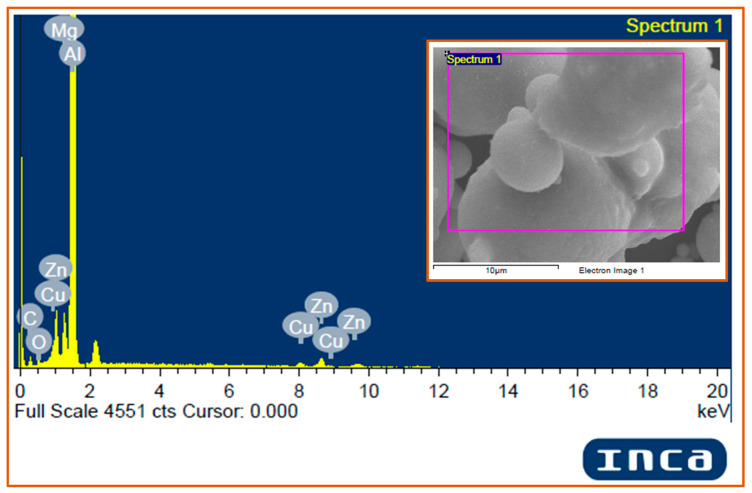
EDS Spectrum of the matrix Al 7075.

**Figure 9:**
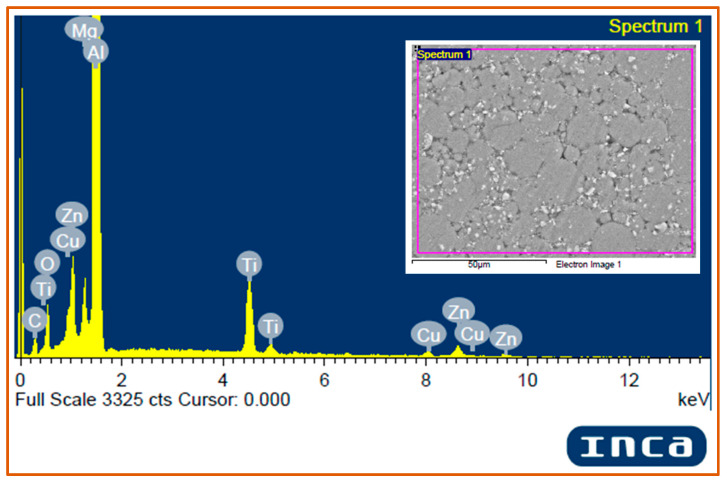
EDS Spectrum of the hybrid composites (Al 7075 + 3 wt.% TiC + 1 wt.% Gr).

The energy dispersive spectroscopy (EDS) analysis of the matrix powder Al 7075 (Figure 8) confirmed the presence of all the major alloying elements (Zn, Mg, and Cu) in the Al 7075 alloy powder, however, alloying elements of less than 0.1 wt.% are not detected in the image due to their low content. It is also observed that a small peak of oxygen is present in the Al 7075 EDS spectrum. The EDS analysis of hybrid composites (Al 7075 + 3 wt.% TiC + 1 wt.% Gr) was done to validate the presence of the Al 7075 matrix and TiC and graphite reinforcements within the developed hybrid composites (Figure 9). It is observed that the spectrum of Al, Ti, and C peaks confirms the presence of Al, TiC, and graphite in the sintered hybrid composite samples.

It is observed that the spectrum of Al, Ti, and C peaks confirms the presence of Al, TiC, and graphite in the sintered hybrid composite samples. The peaks of alloying elements Cu, Zn, and Mg were distinctly detected in the hybrid composite sample. However, oxygen peaks were also observed and are attributed to the formation of oxides during the sintering process. The respective composition of alloying elements are presented in Table 5.

#### 3.2.4. Sintered Density, Porosity, and Densification Factor

**Figure 10:**
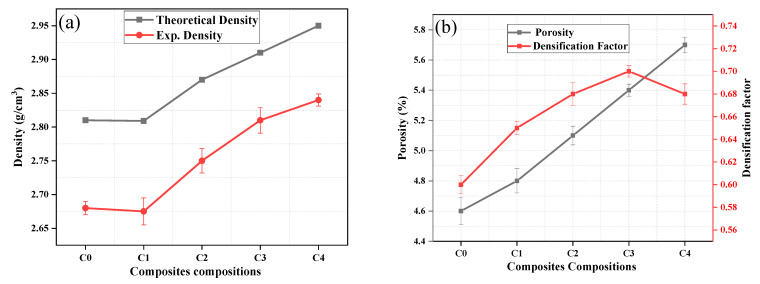
Plot for (**a**) variation of theoretical and sintered density with addition of reinforcements; (**b**) variation of porosity and densification factor as a function of wt.% of reinforcements.

The variation of theoretical and sintered density of Al 7075/TiC/Gr composites is depicted in Figure 10a. The theoretical density (TD) signifies the density of composites with no cavities, i.e., the highest densification in the theoretical state, it is substantially higher than the experimental density. It is observed that the theoretical density of Al 7075/graphite composites (sample C1) slightly decreases as compared to neat aluminum (sample C0). The calculation of TD of composites has been done by the rule of mixtures, and the decline in the theoretical density of composite is attributed to the lower density of graphite as compared to aluminum. It is clear from the theoretical density curve (Figure 10a) that the addition of TiC particles to the aluminum matrix improves the density of the hybrid composites (samples C2, C3, and C4) because the TiC particles possess higher density than the aluminum matrix. An approximate linear drop in the experimental densities is observed (Figure 10a), the decrease in the experimental density is attributed to the presence of pores. Similar findings were observed in the literature [51].

Porosity progression with the addition of TiC is portrayed in Figure 10b. As expected, the porosity increases with the rise of TiC wt.%. High thermal expansion divergence amongst the Al 7075 matrix and the TiC, and the development of agglomerated particles are possibly responsible for the progress of porosity. The densification factor indicates the densification process during the sintering of the composite samples. It is evident from Figure 10b that the densification of the compacts changes as the content of TiC varies. It can be observed that the addition of TiC increases the densification factor of the composites because of the compressibility.

**Figure 11:**
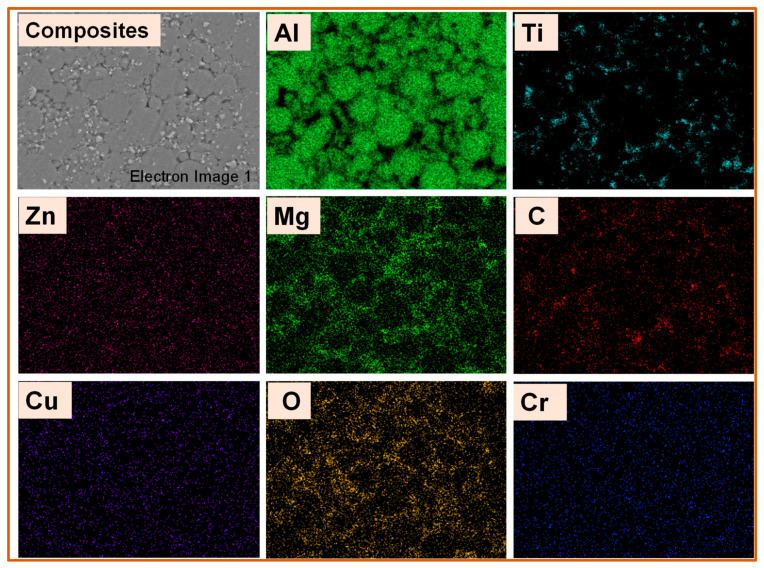
Elemental maps of Al 7075 + 3 wt.% TiC + 1 wt.% Gr hybrid composites.

The balanced distribution of TiC and graphite (carbon) in the matrix Al 7075 can be seen from the elemental mapping analysis (Figure 11). As a result, the EDS and elemental mapping analyses confirm that the composite was successfully synthesized with an even and random distribution of TiC and graphite in the Al 7075 matrix. The presence of oxygen is attributed to the formation of Al-oxides during blending and sintering.

### 3.3. Response Surface Model Development

To investigate the effect of process parameters (i.e., TiC wt.%, compaction pressure, and sintering temperature) on the density and microhardness, a response surface methodology was adopted. The Box-Behnken design (BBD) interface of RSM has been used for the experimental design. Table 4 presents a design of the experiment for three variables with coded values and experimentally obtained responses value from conducting a total of 17 statistically designed experiments. The next stage is to use the most extensively used second-order polynomial response surface function to take the datasets of independent variables (x) and the corresponding output (R) to evaluate the coefficients of the model, identify interactions amongst those independent variables, ascertain the curvature, optimize the process, and find a suitable model to predict the density and microhardness. The model utilized in this investigation is the quadratic polynomial expressed by the following equation:

(3) $$\;R=\;\beta o+\overset{3}{\underset{i=1}{\sum }}\beta_{i}x_{i}+\overset{2}{\underset{i=1}{\sum }}\overset{3}{\underset{i=j+1}{\sum }}\beta_{\mathrm{ij}}x_{i}x_{j}+\overset{3}{\underset{i=1}{\sum }}\beta_{\mathrm{ii}}x_{i}^{2}$$

where R is the response value, βo denotes the fixed response value, $\beta_{i,}$ $\beta_{\mathrm{ij},\;}{\;\mathrm{and}\;\beta }_{\mathrm{ii}}$ represents the coefficients of linear, interactive, and quadratic parameters, respectively. ANOVA is a statistical analysis method for analyzing experimental data that evaluates the proportion of the impact of one or more factors on overall variation [52], applying ANOVA is the most consistent approach to assess the quality of a fitted model. 

### 3.4. Effect of Process Parameters on Characteristics of Synthesized Composites

#### 3.4.1. Effect of Process Variables on Microhardness

The relationship between the response (microhardness) and independent variables was evaluated by Equation (2). The equation is produced in terms of coded factors, and it predicts the response for presented levels of all the factors. The developed RSM model for microhardness was evaluated at a 95% confidence interval for its algorithmic significance [53]. The coded equation analyzes the coefficients of the variables to establish the comparative effect of parameters. The reciprocal and direct relationships of respective parameters with the response surface are represented by the negative and positive signs in the equation, respectively:

Microhardness = +73.1 + 4.775 × A + 1.0875 × B + 3.4125 × C + 2.325 × AB + 1.98 × AC − 0.45 × BC − 4.25 × A^2^ − 0.325 × B^2^ + 2.73 × C^2^ (4)

Table 6 summarizes the findings of ANOVA and the F-Test utilized for evaluating the statistical significance of the aforesaid quadratic model. The modest Fisher’s F-Test value (F = 24.46) with a lower possibility value (*p*-value) > F (0.0002) demonstrates the superior statistical significance of the regression model to signify the real relationship between the achieved experimental data of microhardness and three variables with a fewer chance. If the probability values (*p*-values) of the process variables (i.e., individual, interaction, and quadratic terms) in the design model of a specific response of interest are found to be less than 0.05, then they are considered statistically considerable.

The statistically significant terms are observed to be A (TiC content), C (sintering temperature), AB (interaction of TiC content and compaction pressure), AC (interaction of TiC content and sintering temperature), A^2^ (quadratic term of TiC content), and B^2^ (quadratic term of compaction pressure). The relatively high influence of reinforcement content on the microhardness of the analyzed specimens is consistent with a few previous investigations [54]. The lack of fit F-value of 1.19 implies the lack of fit is not significant relative to the pure error. There is a 41.93% chance that a lack of fit F-value this large could occur due to noise. Non-significant lack of fit implies that the contribution of the chosen variable is adequately high for producing the full model, and thus it is recommended. Thus, the achieved outcomes are in good agreement with previously reported literature [34,45]. The correlation coefficient (R^2^) of 0.9692 indicates that the model developed is accurate and closely fits the experimental data. The adjusted R^2^ of 0.9296 is reasonably close to the predicted R^2^ of 0.8420. The difference between adjusted R^2^ and predicted R^2^ was obtained to be 0.08, which is inside the acceptable range (<0.2) for a developed model. The predicted R^2^ indicates how well the developed model responds to new observations and whether the model is simple or complicated. As a result, in statistics, adjusted R^2^ and predicted R^2^ are more desirable attributes for the goodness of fit. In the present study, all the R^2^ characteristics were near to 1, implying that the data and models generated were incredibly significant.

#### 3.4.2. Effect of Process Variables on Density

Analysis of variance (ANOVA) and a lack of fit test (LOF) was performed to justify the model’s adequacy. According to results of the ANOVA analysis (Table 7), the model regression coefficient of determination (R^2^) of 0.9295 for sintered density was in good agreement with the experimental outcomes, indicating that the model can explain 92.95% of the variability, leaving 7.05% residual variability for output (density). The design model’s final quadratic equation (Equation (3)) with the three independent variables and dependent response (density) can be represented as:

Density = 2.846 + 0.0325 × A + 0.01125 × B + 0.04875 × C − 7.13 × 10^−16^ × AB + 0.03 × AC + 0.0075 × BC − 0.05675 × A^2^ − 0.04425 × B^2^ + 0.03075 × C^2^ (5)

From Table 7 it is observed that *p*-value for the model is less than 0.05, indicating the model is significant. Also, A, C, AC, A², B², and C² are the significant model terms. The lack of fit F-value equal to 0.6735 implies that the LOF is low as compared to the absolute error, hence non-significant. A non-significant LOF value indicates that the specified variable’s involvement is sufficient for generating the full model. The ANOVA results revealed that the model is vastly significant (*p*-value < 0.002). In previously reported research, a high coefficient of determination closer to 1 is desirable.

#### 3.4.3. Assessment of Actual and Predicted Responses (Density and Microhardness)

**Figure 12:**
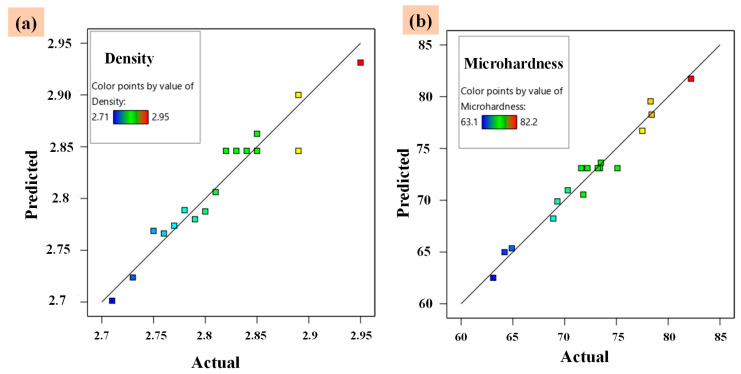
The actual vs. Predicted plots of two responses obtained from RSM. (**a**) Sintered density, (**b**)Microhardness.

To verify the adequacy of the developed model, the predicted versus actual responses were plotted. The RSM model’s effectiveness for the responses was tested by putting the data in the developed model. As illustrated in Figure 12, the experimental results are compared with the predicted data derived from the analysis. The predicted value points have been observed to be evenly dispersed, close to the parity line, and showed a straight-line fit. This adds credence to the model’s robustness. The proposed quadratic models for the responses performed effectively with an insignificant lack of fit and had no issues in predicting the response values, as evidenced by the minimum spread of the predicted data points in both cases [55].

### 3.5. Response Surfaces Interaction of RSM Model

#### 3.5.1. Effect of the Parameters and Their Interaction on Density

**Figure 13:**
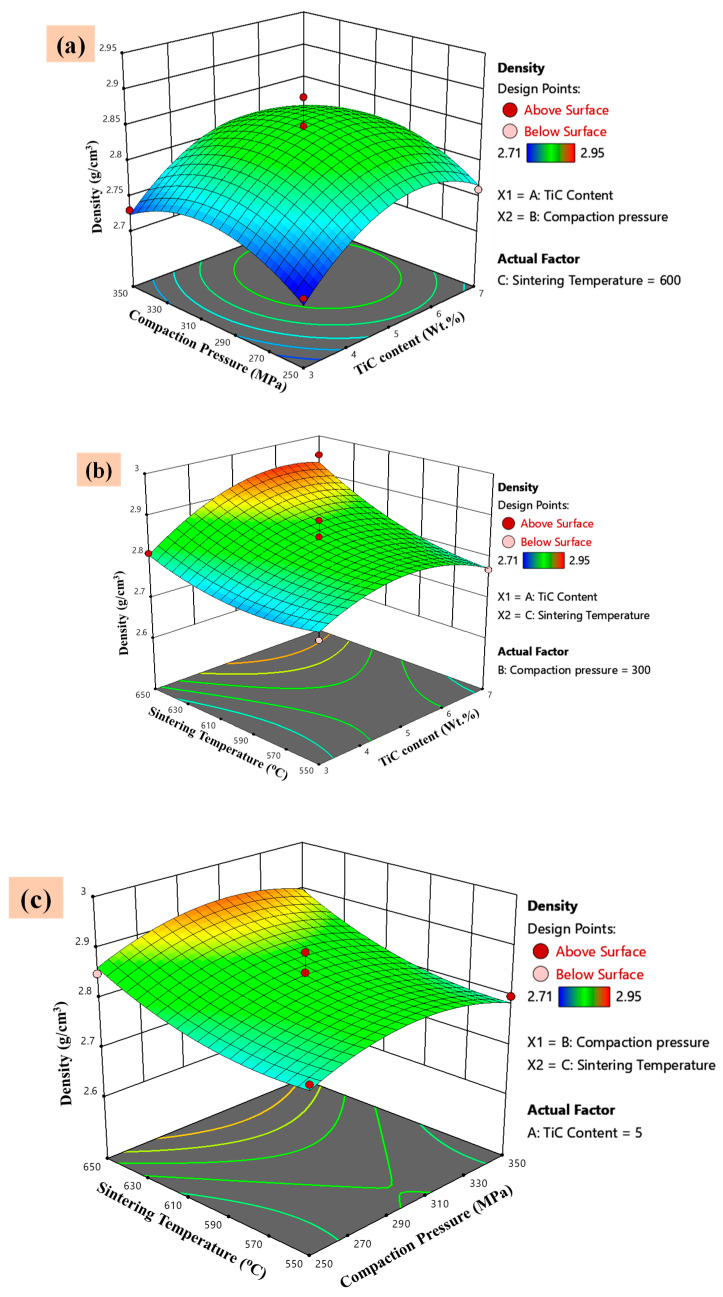
3D-surface plots of the significant interacting factors with the density response. (**a**) Compaction pressure vs. TiC content, (**b**) Sintering temperature vs. TiC Content, (**c**) Sintering temperature vs. Compaction pressure.

The regression findings for the density response are shown graphically in Figure 13a–c, as a three-dimensional (3D) response surface plot. The effects of compaction pressure and reinforcement content (TiC wt.%) on the density, as well as the interaction between these parameters, are portrayed in Figure 13a. The findings indicate that TiC wt.% had a significant effect on the density of the synthesized composites. This effect is attributed to the addition and Well distribution of high-density reinforcement (TiC particles) within the lighter matrix Al 7075. Several studies have also revealed that the content of the TiC is directly proportional to the density of the metal matrix composites [51,56,57]. Remarkably, no significant interaction was witnessed between the compaction pressure and TiC content (*p* = 1, Figure 13a).

A 3D surface plot encapsulating the effects of the sintering temperature and TiC wt. % on the sintered density is exhibited in Figure 13b. The density of the synthesized composites also increased with increasing sintering temperature from 500 to 650 °C (Figure 13b). The increase in sintered density with sintering temperature is attributed to the reduction in porosities between the matrix and the reinforcements. The maximum density increment in composites as compared to the Al 7075 matrix was achieved (4.2%) at 650 °C sintering temperature and 7 wt.% TiC contents. Previous investigations have observed similar findings [58,59]. Sintering temperature also improves the other mechanical properties (tensile strength, fracture toughness, microhardness) of various composites. Furthermore, the 3D graph (Figure 13b) depicts that the sintered density was considerably influenced by both factors. However, the sintering temperature had a greater effect than the TiC content. A significant interaction was observed between the sintering temperature and titanium carbide (*p* = 0.0478, Figure 13b)

The effects of compaction pressure and sintering temperature on the sintered density are shown in Figure 13c. The sintering temperature affects more as compared to the compaction pressure at higher reinforcement content. This is attributed to the removal of porosities from composites, at higher sintering temperatures. The combined effect of the compaction pressure and sintering temperature were insignificant as (*p* > 0.05). The response surface interactions acquired are in agreement with the ANOVA results and were observed to be coherent with the literature [54,60].

#### 3.5.2. Effect of the Parameters and Their Interaction on Microhardness

**Figure 14:**
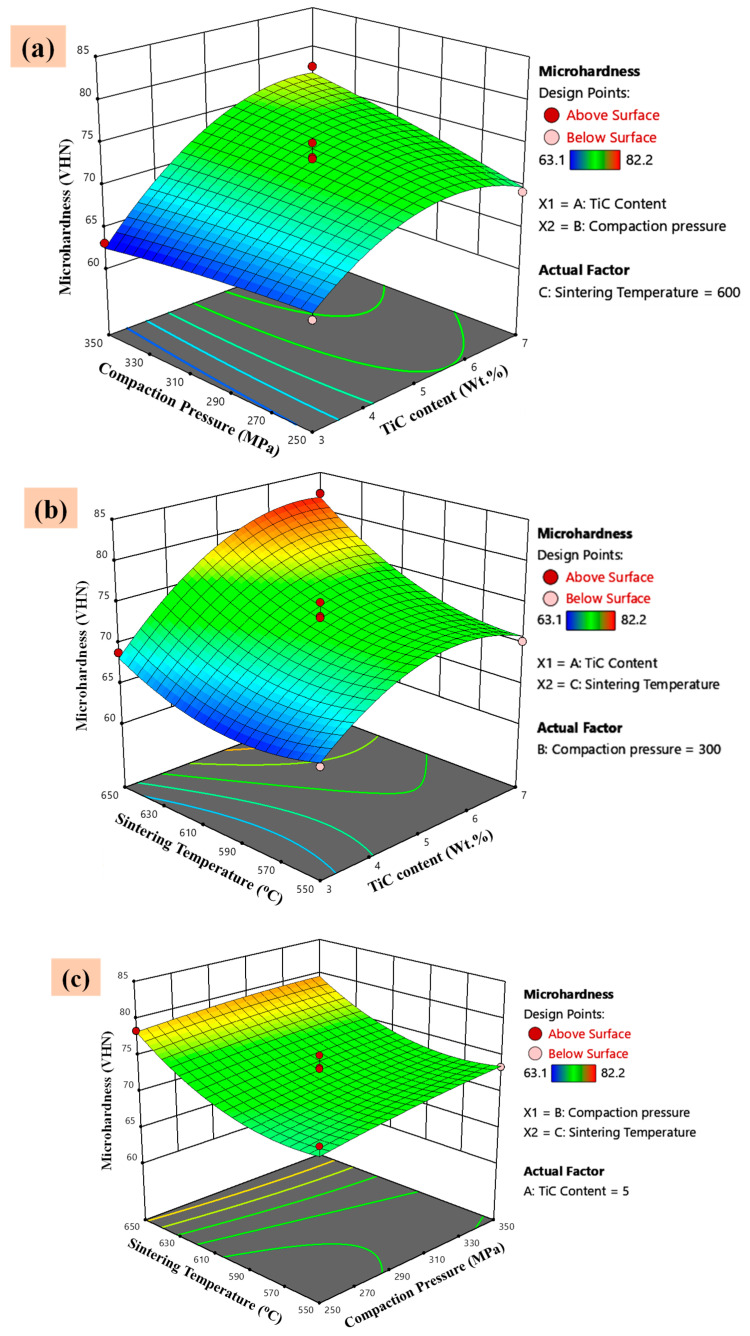
3D-surface plots of the significant interacting factors with response microhardness. (**a**) Compaction pressure vs. TiC content, (**b**) Sintering temperature vs. TiC Content, (**c**) Sintering temperature vs. Compaction pressure.

The response surface plots for the variation of microhardness with sintering temperature, compaction pressure, and TiC wt.% content are presented in Figure 14. The TiC content, sintering temperature, and compaction pressure have a positive effect on the microhardness as shown in Figure 14, the reinforcement (TiC) content is the more effective parameter. The surfaces of the output better signify the capability of an individual parameter to affect the microhardness. It is observed from the 3D response surface plots of Figure 14a,b, TiC concentration is revealed to have the most significant effect on the microhardness of the developed composites, followed by the sintering temperature and compaction pressure. Experimental investigations performed by many researchers have concluded that the TiC concentration is directly proportional to the microhardness of Al alloy-based composites [45,47]. Hence, the results achieved in the present study are analogous to previous investigations. Figure 14a represents the interaction between the TiC content and compaction pressure, and it is found to be significant (*p* < 0.05) as observed from ANOVA (Table 6).

Sintering temperature has a significant effect on the microhardness of synthesized composites. The effect of sintering temperature on the response is presented in Figure 14b,c as a 3D surface plot. It is observed that with the increase in temperature the microhardness increases. This is attributed to the more effective activation of the sintering mechanism and hence the density of the composites is improved and consequently the microhardness increases. Thus, the sintering temperature is the most significant parameter as the *p*-value is less than 0.05 (Table 6). The interaction between sintering temperature and TiC concentration, presented in Figure 14b, is also significant (*p* < 0.05).

The effect of the compaction pressure and sintering temperature on the microhardness is shown in Figure 14c. The compaction pressure is the least significant parameter among all three process variables. Thus, the interaction between compaction pressure and sintering temperature is not significant (*p* > 0.05).

### 3.6. Desirability Based Response Optimization

An optimization process was done after an effective analysis of the response model. The optimization of process parameters for the development of hybrid composites with improved mechanical properties has been carried out through the RSM technique of the Design-Expert 12 software (Stat-Ease, Inc. Minneapolis, MN, USA, v12). To achieve optimum input parameters to maximize the responses, the ranges, and goals of input variables viz. TiC concentration, compaction pressure, sintering temperature, and the output parameters density and microhardness are illustrated in Table 8.

**Figure 15:**
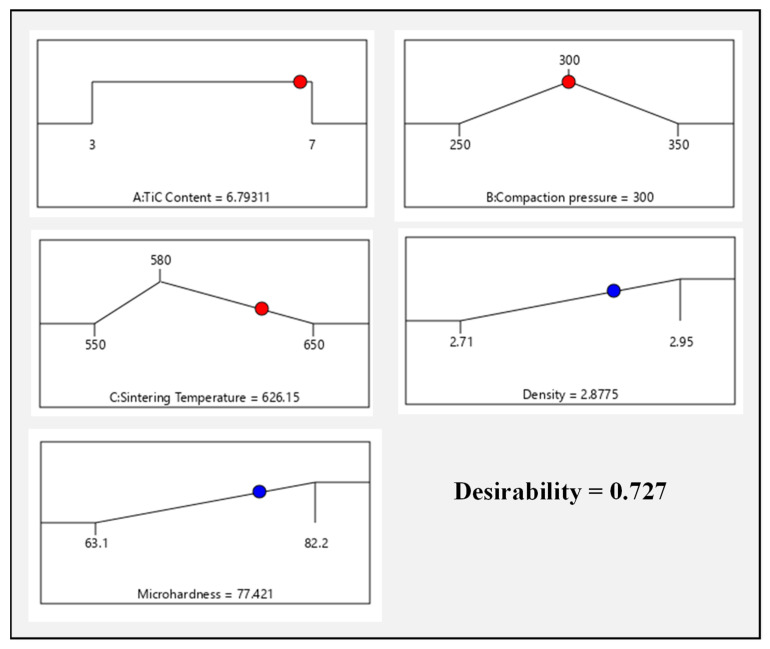
Ramp function graph showing the optimal value of each variable and combined desirability function.

A set of five optimal solutions is derived for the given input constraints for density and microhardness using the Design-Expert software as given in Table 9. The optimal solution for the desired response is chosen from a range of conditions with the highest desirability value. Table 9 depicts the ideal set of conditions with the highest desirability function. As it is observed from the ramp function graph (Figure 15), the TiC concentration of 6.79 wt.%, compaction pressure of 300 MPa, the sintering temperature of 626 °C are the optimum process variables used to get the enhanced density 2.87 g/cm^3^ and microhardness 77.42 VHN of the synthesized composites. The desirability, in general, varies from 0 to 1 depending upon the closeness of the output to the set target. 

**Figure 16:**
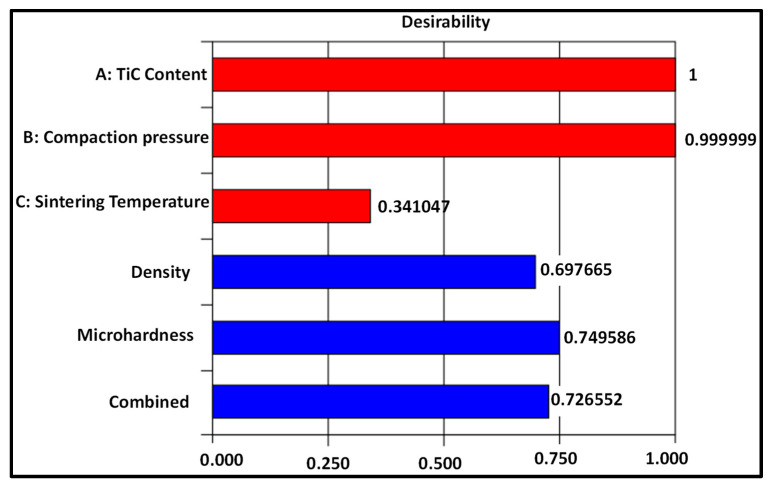
Bar graph of desirability optimization of each parameter.

The ramp function and bar graphs are shown in Figure 15 and Figure 16, illustrating the desirability of the output responses. For each response characteristic, the dot on each ramp depicts the variable setting or response predictions. The size of the dot indicates how desirable it is. Because the weight for each variable has been set to one, a linear ramp function was produced between the minimal value and the goal or the maximum value and the goal.

The overall desirability function of each response is illustrated by a bar graph (Figure 16) which displays how well each variable meets the requirement; values close to one are considered satisfactory. The desirability of individual responses was obtained by employing the desirability estimation profiler. By using a separate model for each response, this desirability prediction function can perform simultaneous optimization of many solutions. The desirability function (0.727) reflects the good suitability of process variables to achieve a better response within the given preset target, hence it is confirmed that the response surface methodology is a useful technique for determining the optimized solutions for a specific problem.

**Figure 17:**
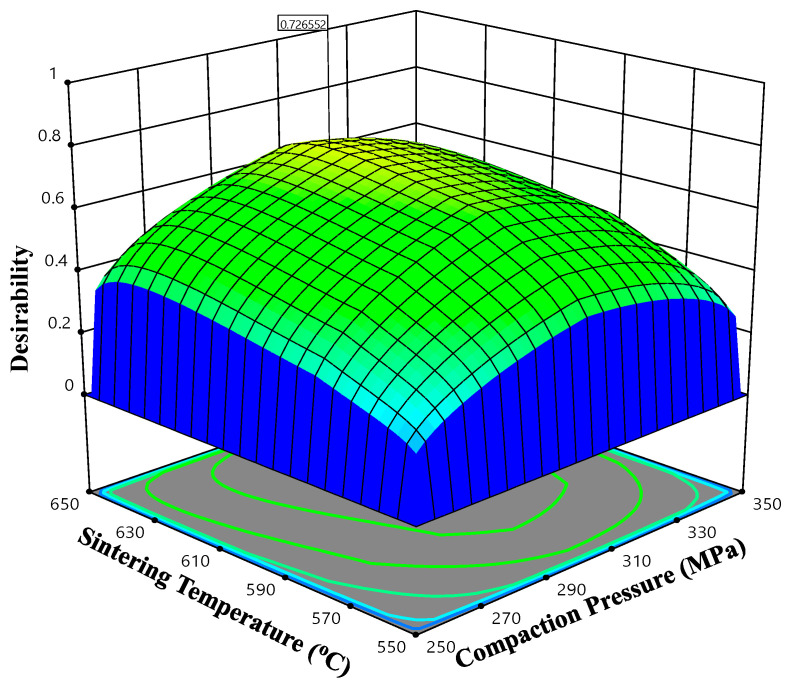
Combined 3-D desirability plot obtained from the multi-response desirability analysis.

Figure 17 demonstrates the influence of input process factors on the desirability, as well as overall 3-D and contour plots with TiC content as the actual factor. The near-optimal zone was displayed on the top region of the contour plot.

It is evident from the plot that the optimum conditions are desired at the compaction pressure range of 290–310 MPa and sintering temperature range of 610–630 °C at the constant TiC 6.79 wt.% with desirability varying between 0.625–0.727. This shows that the optimum parameters (i.e., maximum density and microhardness) might be attained within the parameters mentioned above

### 3.7. Experimental Verification on Optimized Conditions of Process Variables

For additional validation of the developed models, a confirmation test was carried out by adding the optimized input parameters. Table 10 presents the comparisons between the experimental and predicted values. It is observed that the experimental values are reasonably close to the predicted values, with just a minimum error of below 3%. This shows that the model can effectively predict the microhardness and density values. Therefore, it can be established that the newly developed model is a satisfactorily accurate model for predicting the response and can be applied to simulate the density and microhardness for any newly synthesized formulation under similar experimental conditions.

## 4. Conclusions

The current study explores the synthesis, microstructural characterization, modeling, and optimization of Al 7075-Gr 1 wt.%-TiC x wt.%, TiC (x = 3, 5, and 7%) sintered hybrid composites, produced via a powder metallurgy technique. The microstructural characteristics of base materials and synthesized composites were investigated by optical microscopy, scanning electron microscopy (SEM), X-ray diffraction (XRD) and energy dispersive X-ray spectroscopy (EDS) elemental mapping. The effect of input process variables (i.e., TiC concentration, sintering temperature, compaction pressure) on sintered density and microhardness characteristics has been examined. For the experimental design, modeling, and optimization of processing parameters, the RSM modeling approach was utilized. The produced hybrid composites have superior density and microhardness as compared to the matrix Al 7075.

The presence of TiC is the most significant parameter for enhancement in density and microhardness. The EDS analysis and elemental mapping verified the several alloying elements in the base matrix Al 7075 and synthesized Al 7075/TiC/Gr hybrid composite. The optical microscopy and FESEM analysis of the synthesized samples confirmed the proper dispersion of reinforcements within the matrix. The grain refinement improves with increasing content of TiC particles. An effective model has been successfully developed by RSM, that correlates several environmental factors with density and microhardness. The developed models have excellent agreement among experimental and predicted values. The quadratic model proposed by the BBD tool of RSM is utilized to optimize the process variables. The AVONA table generated and found the model to be significant for both the responses. A set of 17 experiments were performed for optimization study at different combinations of process variables. The developed model accurately predicted the optimal conditions for high density and microhardness. A maximum sintered density of 2.87 g/cm^3^, and microhardness of 77.41 VHN were achieved using the optimized conditions of a TiC content of 6.79 wt. %, a sintering temperature of 626.13 °C, and compaction pressure of 300 MPa. All the selected process variables were shown to have a significant effect on both the responses, although the TiC concentration and sintering temperature had the most noticeable effects. In a nutshell, the Al 7075–6.7 wt.% TiC–1 wt.% Gr hybrid composite has shown to be a highly effective material for potential aerospace applications. The research outcomes along with RSM models and multi-response optimization will provide a useful guidance in selecting process parameters and the result will be a good technical database for the aerospace, and military applications in fabrication and performance evaluation aspects.

## Figures and Tables

**Table 1:** Al 7075 alloy compositions.

Elements	Si	Cr	Mn	Fe	Cu	Mg	Ai	Zn	Al
**wt.%**	0.087	0.185	0.08	0.092	1.56	2.31	0.05	5.72	Bal.

**Table 2:** Compositions of synthesizing Al/Gr/TiC composites.

S. No	Samples ID	The Weight Percentage of Al Matrix and Reinforcements Element		
		Al	TiC	Gr
1.	C0	100	0	0
2.	C1	99	0	1
3.	C2	96	3	1
4.	C3	94	5	1
5.	C4	92	7	1

**Table 3:** Independent variables DOE ranges and levels.

Factor	Name	Units	Ranges and Levels		
			−1	0	+1
A	TiC Concentration	wt.%	3	5	7
B	Compaction Pressure	MPa	250	300	350
C	Sintering Temperature	°C	550	600	650

**Table 4:** Matrix of design (Box-Behnken method) with experimental values.

Experimental Run	TiC Concentration (wt.%)	Compaction Pressure (MPa)	Sintering Temperature (°C)	Microhardness (VHN)	Density (kg/m^3^)
1	−1	−1	0	64.2	2.71
2	+1	−1	0	69.3	2.76
8	−1	+1	0	63.1	2.73
3	+1	+1	0	77.5	2.78
7	−1	0	−1	57.9	2.75
9	+1	0	−1	60.3	2.77
13	−1	0	+1	71.9	2.81
17	+1	0	+1	82.2	2.95
12	0	−1	−1	68.2	2.79
4	0	+1	−1	73.5	2.80
6	0	−1	+1	74.4	2.85
10	0	+1	+1	78.3	2.89
11	0	0	0	76.2	2.83
5	0	0	0	69.4	2.85
14	0	0	0	61.4	2.89
16	0	0	0	64.2	2.82
15	0	0	0	65.1	2.84

**Table 5:** Elements present in (Al 7075 + 3 wt.% TiC + 1 wt.% Gr) hybrid composites.

Elements	Weight%	Atomic%
C	13.65	22.60
O	9.50	13.84
Mg	1.65	1.58
Al	66.35	57.32
Ti	3.90	2.87
Cu	1.26	0.46
Zn	3.69	1.33
Total	100	100

**Table 6:** ANOVA table for microhardness acquired from (BBD) RSM.

Response	Source	Sum of Squares	DOF	Mean Square	F-Value	*p*-Value	
Micro hardness	Model	426.24	9	47.36	24.46	0.0002	significant
	A-TiC Content	182.41	1	182.41	94.21	<0.0001	
	B-Compaction Pressure	9.46	1	9.46	4.89	0.0627	
	C-Sintering Temperature	93.16	1	93.16	48.12	0.0002	
	AB	21.62	1	21.62	11.17	0.0124	
	AC	15.60	1	15.60	8.06	0.0251	
	BC	0.8100	1	0.8100	0.4184	0.5384	
	A²	76.05	1	76.05	39.28	0.0004	
	B²	0.4447	1	0.4447	0.2297	0.6463	
	C²	31.27	1	31.27	16.15	0.0051	
	Residual	13.55	7	1.94			
	Lack of Fit	6.39	3	2.13	1.19	0.4193	not significant
	Pure Error	7.16	4	1.79			
	Cor Total	439.80	16				

R^2^ = 0.9692, Adj. R^2^ = 0.9296 and Pred. R^2^ = 0.8420.

**Table 7:** ANOVA table for density obtained from (BBD) RSM.

Response	Source	Sum of Squares	DOF	Mean Square	F-Value	*p*-Value	
Density	Model	0.0580	9	0.0064	10.26	0.0028	significant
	A-TiC Content	0.0085	1	0.0085	13.46	0.0080	
	B-Compaction pressure	0.0010	1	0.0010	1.61	0.2447	
	C-Sintering Temperature	0.0190	1	0.0190	30.28	0.0009	
	AB	0.0000	1	0.0000	0.0000	1.0000	
	AC	0.0036	1	0.0036	5.73	0.0478	
	BC	0.0002	1	0.0002	0.3584	0.5683	
	A²	0.0136	1	0.0136	21.60	0.0023	
	B²	0.0082	1	0.0082	13.13	0.0085	
	C²	0.0040	1	0.0040	6.34	0.0399	
	Residual	0.0044	7	0.0006			
	Lack of Fit	0.0015	3	0.0005	0.6735	0.6118	not significant
	Pure Error	0.0029	4	0.0007			
	Cor Total	0.0624	16				

R^2^ = 0.9295, Adj. R^2^ = 0.8389 and Pred. R^2^ = 0.7410.

**Table 8:** Pre-set goals of input parameters and responses for desirability.

Parameters	Pre-Set Goal	Lower Limit	Upper Limit	Level of Importance
TiC Content	is in range	3	7	3
Compaction pressure	Target = 300	250	350	3
Sintering temperature	Target = 580	550	650	3
Microhardness	Maximize	63.1	82.2	5
Density	Maximize	2.71	2.95	5

**Table 9:** Numerical optimization solutions with desirability.

No of Solutions	TiC (wt.%)	Compaction Pressure (MPa)	Sintering Temperature (°C)	Density (g/cm^3^)	Micro-Hardness (VHN)	Desirability
1	6.793	300.000	626.130	2.877	77.418	0.727
2	6.761	300.001	625.606	2.877	77.362	0.727
3	6.846	300.001	626.562	2.877	77.437	0.726
4	6.740	300.001	625.536	2.878	77.369	0.726
5	6.739	300.001	624.397	2.875	77.191	0.726

**Table 10:** Experimental verification results comparison.

Run	Microhardness			Density		
	Experimental	Predicted	% Error	Experimental	Predicted	% Error
1	76.38	77.42	1.36	2.79	2.87	2.86
2	75.82	77.42	2.19	2.85	2.87	1.75
3	74.23	77.42	4.2	2.83	2.87	1.41
4	75.47	77.42	2.58	2.81	2.87	2.13
Mean			2.58			2.03

## Data Availability

The data is available on request from the corresponding author.

## References

[1] Agarwal A.K., Mustafi N.N. (2021). Real-world automotive emissions: Monitoring methodologies, and control measures. Renew. Sustain. Energy Rev..

[2] Voelcker J. (2015). 1.2 Billion Vehicles On World’s Roads Now, 2 Billion By 2035: Report. Green Car Rep..

[3] Brinson L.C. (2012). How Much Air Pollution Comes from Cars?.

[4] Bin Muhamad M.R., Raja S., Jamaludin M.F., Yusof F., Morisada Y., Suga T., Fujii H. (2021). Enhancements on dissimilar friction stir welding between AZ31 and SPHC mild steel with Al-Mg as powder additives. J. Manuf. Sci. Eng. Trans. ASME.

[5] Alam M.A., Sapuan S.M., Ya H.H., Hussain P.B., Azeem M., Ilyas R.A. (2021). Application of biocomposites in automotive components: A review. Biocomposite and Synthetic Composites for Automotive Applications.

[6] Wang Z.J., Liu S., Qiu Z.X., Sun H.Y., Liu W.C. (2020). Applied Surface Science First-principles calculations on the interface of the Al/TiC aluminum matrix composites. Appl. Surf. Sci..

[7] Leszczyńska-madej B., Garbiec D., Madej M. (2020). Effect of sintering temperature on microstructure and selected properties of spark plasma sintered Al-SiC composites. Vacuum.

[8] Shaikh M.B.N., Arif S., Aziz T., Waseem A., Shaikh M.A.N., Ali M. (2019). Microstructural, mechanical and tribological behaviour of powder metallurgy processed SiC and RHA reinforced Al-based composites. Surf. Interfaces.

[9] Dursun T., Soutis C. (2014). Recent developments in advanced aircraft aluminium alloys. Mater. Des..

[10] Alam M.T., Ansari A.H., Arif S., Alam M.N. (2017). Mechanical properties and morphology of aluminium metal matrix nanocomposites-stir cast products. Adv. Mater. Process. Technol..

[11] Alam M.A., Ya H.H., Ahmad A., Yusuf M., Azeem M., Masood F. (2021). Influence of aluminum addition on the mechanical properties of brass/Al composites fabricated by stir casting. Mater. Today Proc..

[12] Qiu F., Tong H.T., Gao Y.Y., Zou Q., Dong B.X., Li Q., Chu J.G., Chang F., Shu S.L., Jiang Q.C. (2018). Microstructures and compressive properties of Al matrix composites reinforced with bimodal hybrid in-situ nano-/micro-sized TiC particles. Materials.

[13] Raja S., Muhamad M.R., Jamaludin M.F., Yusof F. (2020). A review on nanomaterials reinforcement in friction stir welding. J. Mater. Res. Technol..

[14] Gangil N., Noor A., Maheshwari S. (2017). Aluminium based in-situ composite fabrication through friction stir processing: A review. J. Alloys Compd..

[15] Chak V., Chattopadhyay H., Dora T.L. (2020). A review on fabrication methods, reinforcements and mechanical properties of aluminum matrix composites. J. Manuf. Process..

[16] Moustafa E.B., Mosleh A.O. (2020). Effect of (Ti–B) modifier elements and FSP on 5052 aluminum alloy. J. Alloys Compd..

[17] Cabeza M., Feijoo I., Merino P., Pena G., Pérez M.C., Cruz S., Rey P. (2017). Effect of high energy ball milling on the morphology, microstructure and properties of nano-sized TiC particle-reinforced 6005A aluminium alloy matrix composite. Powder Technol..

[18] Popov V.A., Burghammer M., Rosenthal M., Kotov A. (2018). In situ synthesis of TiC nano-reinforcements in aluminum matrix composites during mechanical alloying. Compos. Part B.

[19] Omrani E., Moghadam A.D., Menezes P.L., Rohatgi P.K. (2015). Influences of graphite reinforcement on the tribological properties of self-lubricating aluminum matrix composites for green tribology, sustainability, and energy efficiency—A review. Int. J. Adv. Manuf. Technol..

[20] Lu Y., Watanabe M., Miyata R., Nakamura J., Yamada J., Kato H., Yoshimi K. (2020). Materials Science & Engineering A Microstructures and mechanical properties of TiC-particulate-reinforced Ti—Mo—Al intermetallic matrix composites. Mater. Sci. Eng. A.

[21] Mahanta S., Chandrasekaran M., Samanta S., Arunachalam R. (2019). Multi-response ANN modelling and analysis on sliding wear behavior of Al7075/B4C/fly ash hybrid nanocomposites. Mater. Res. Express.

[22] Haider K., Alam M.A., Redhewal A., Saxena V. (2015). Investigation of Mechanical Properties of Aluminium Based Metal Matrix Composites Reinforced with Sic & Al_2_O_3_. Int. J. Eng. Res. Appl..

[23] Nassar A.E., Nassar E.E. (2017). Properties of aluminum matrix Nano composites prepared by powder metallurgy processing. J. King Saud Univ. Sci..

[24] Esmati M., Sharifi H., Raesi M., Atrian A., Rajaee A. (2019). Investigation into thermal expansion coefficient, thermal conductivity and thermal stability of Al-graphite composite prepared by powder metallurgy. J. Alloys Compd..

[25] Yu H., Zhang S.Q., Xia J.H., Su Q., Ma B.C., Wu J.H., Zhou J.X., Wang X.T., Hu L.X. (2021). Microstructural evolution, mechanical and physical properties of graphene reinforced aluminum composites fabricated via powder metallurgy. Mater. Sci. Eng. A.

[26] Halil K., Ismail O.I., Sibel D., Ramazan Ç. (2019). Wear and mechanical properties of Al6061/SiC/B4C hybrid composites produced with powder metallurgy. J. Mater. Res. Technol..

[27] Sivasankaran S., Ramkumar K.R., Al-Mufadi F.A., Irfan O.M. (2021). Effect of TiB2/Gr Hybrid Reinforcements in Al 7075 Matrix on Sliding Wear Behavior Analyzed by Response Surface Methodology. Met. Mater. Int..

[28] Kumar R., Chauhan S. (2015). Study on surface roughness measurement for turning of Al 7075/10/SiCp and Al 7075 hybrid composites by using response surface methodology (RSM) and artificial neural networking (ANN). Meas. J. Int. Meas. Confed..

[29] Yusuf M., Farooqi A.S., Alam M.A., Keong L.K., Hellgardt K., Abdullah B. (2021). Response surface optimization of syngas production from greenhouse gases via DRM over high performance Ni–W catalyst. Int. J. Hydrogen Energy.

[30] Khuri A.I., Mukhopadhyay S. (2010). Response surface methodology. Wiley Interdiscip. Rev. Comput. Stat..

[31] Masood F., Nallagownden P., Elamvazuthi I., Akhter J., Alam M.A. (2021). A New Approach for Design Optimization and Parametric Analysis of Symmetric Compound Parabolic Concentrator for Photovoltaic Applications. Sustainability.

[32] Yusuf M., Farooqi A.S., Al-Kahtani A.A., Ubaidullah M., Alam M.A., Keong L.K., Hellgardt K., Abdullah B. (2021). Syngas production from greenhouse gases using Ni–W bimetallic catalyst via dry methane reforming: Effect of W addition. Int. J. Hydrogen Energy.

[33] Alam M.A., Ya H.H., Azeem M., Hussain P., Bin Salit M.S., Khan R., Arif S., Ansari A.H. (2020). Modelling and optimisation of hardness behaviour of sintered Al/SiC composites using RSM and ANN: A comparative study. J. Mater. Res. Technol..

[34] Taherzadeh Mousavian R., Behnamfard S., Heidarzadeh A., Taherkhani K., Azari Khosroshahi R., Brabazon D. (2020). Incorporation of SiC Ceramic Nanoparticles into the Aluminum Matrix by a Novel Method: Production of a Metal Matrix Composite. Met. Mater. Int..

[35] El-kady O., Fathy A. (2014). Effect of SiC particle size on the physical and mechanical properties of extruded Al matrix nanocomposites. Mater. Des..

[36] Chand S., Chandrasekhar P. (2020). Influence of B4C/BN on solid particle erosion of Al6061 metal matrix hybrid composites fabricated through powder metallurgy technique. Ceram. Int..

[37] Karbalaei Akbari M., Shirvanimoghaddam K., Hai Z., Zhuiykov S., Khayyam H. (2017). Nano TiB2 and TiO2 reinforced composites: A comparative investigation on strengthening mechanisms and predicting mechanical properties via neural network modeling. Ceram. Int..

[38] Maurya R., Kumar B., Ariharan S., Ramkumar J., Balani K. (2016). Effect of carbonaceous reinforcements on the mechanical and tribological properties of friction stir processed Al6061 alloy. Mater. Des..

[39] Moghanlou F.S., Nekahi S., Vajdi M., Ahmadi Z., Motallebzadeh A., Shokouhimehr A., Shokouhimehr M., Jafargholinejad S., Asl M.S. (2020). Effects of graphite nano-flakes on thermal and microstructural properties of TiB2–SiC composites. Ceram. Int..

[40] Şenel M.C., Gürbüz M., Koç E. (2018). Mechanical and tribological behaviours of aluminium matrix composites reinforced by graphene nanoplatelets. Mater. Sci. Technol..

[41] Naseer A., Ahmad F., Aslam M., Guan B.H., Harun W.S.W., Muhamad N., Raza M.R., German R.M. (2019). A review of processing techniques for graphene-reinforced metal matrix composites. Mater. Manuf. Process..

[42] Fallahdoost H., Nouri A., Azimi A. (2016). Journal of Physics and Chemistry of Solids Dual functions of TiC nanoparticles on tribological performance of Al/graphite composites. J. Phys. Chem. Solids.

[43] Chaker H., Ameur N., Saidi-Bendahou K., Djennas M., Fourmentin S. (2021). Modeling and Box-Behnken design optimization of photocatalytic parameters for efficient removal of dye by lanthanum-doped mesoporous TiO2. J. Environ. Chem. Eng..

[44] Azimi A., Shokuhfar A., Nejadseyfi O. (2015). Mechanically alloyed Al7075—TiC nanocomposite: Powder processing, consolidation and mechanical strength. Mater. Des..

[45] Chen J.K., Huang I.S. (2013). Thermal properties of aluminum-graphite composites by powder metallurgy. Compos. Part B Eng..

[46] Jeyasimman D., Sivasankaran S., Sivaprasad K., Narayanasamy R., Kambali R.S. (2014). An investigation of the synthesis, consolidation and mechanical behaviour of Al 6061 nanocomposites reinforced by TiC via mechanical alloying. Mater. Des..

[47] El-Sayed Seleman M.M., Ahmed M.M.Z., Ataya S. (2018). Microstructure and mechanical properties of hot extruded 6016 aluminum alloy/graphite composites. J. Mater. Sci. Technol..

[48] Arif S., Jamil B., Naim Shaikh M.B., Aziz T., Ansari A.H., Khan M. (2020). Characterization of surface morphology, wear performance and modelling of graphite reinforced aluminium hybrid composites. Eng. Sci. Technol. Int. J..

[49] Oza M.J., Schell K.G., Bucharsky E.C., Laha T., Roy S. (2020). Developing a hybrid Al–SiC-graphite functionally graded composite material for optimum composition and mechanical properties. Mater. Sci. Eng. A.

[50] Mohapatra S., Chaubey A.K., Mishra D.K., Singh S.K. (2015). Fabrication of Al—TiC composites by hot consolidation technique: Its microstructure and mechanical properties. Integr. Med. Res..

[51] Muthukrishnan N., Davim J.P. (2009). Optimization of machining parameters of Al/SiC-MMC with ANOVA and ANN analysis. J. Mater. Process. Technol..

[52] Ahmed I., Ahmad A., Rahaman S.A., Abdul-rani A., Azad M. (2021). Modelling and optimization of microhardness of electroless Ni–P–TiO_2_ composite coating based on machine learning approaches and RSM. J. Mater. Res. Technol..

[53] Reddy P.V., Prasad P.R., Krishnudu D.M., Goud E.V. (2019). An Investigation on Mechanical and Wear Characteristics of Al 6063/TiC Metal Matrix Composites Using RSM. J. Bio Tribo Corros..

[54] Shah M.U.H., Moniruzzaman M., Reddy A.V.B., Talukder M.M.R., Yusup S.B., Goto M. (2020). An environmentally benign ionic liquid based formulation for enhanced oil spill remediation: Optimization of environmental factors. J. Mol. Liq..

[55] Ravi Kumar K., Kiran K., Sreebalaji V.S. (2017). Micro structural characteristics and mechanical behaviour of aluminium matrix composites reinforced with titanium carbide. J. Alloys Compd..

[56] Hadian M., Shahrajabian H., Rafiei M. (2019). Mechanical properties and microstructure of Al/(TiC + TiB2) composite fabricated by spark plasma sintering. Ceram. Int..

[57] Gürbüz M., Can Şenel M., Koç E. (2018). The effect of sintering time, temperature, and graphene addition on the hardness and microstructure of aluminum composites. J. Compos. Mater..

[58] Mishra S.K., Das S.K. (2005). Sintering and microstructural behaviour of SHS produced zirconium diboride powder with the addition of C and TiC. Mater. Lett..

[59] Ashok Kumar R., Devaraju A. (2020). Modeling of Mechanical Properties and High Temperature Wear Behavior of Al7075/SiC/CRS Composite Using RSM. Silicon.

[60] Rahimi M.H., Shayganmanesh M., Noorossana R., Pazhuheian F. (2019). Modelling and optimization of laser engraving qualitative characteristics of Al-SiC composite using response surface methodology and artificial neural networks. Opt. Laser Technol..

